# An unusual presentation of a swollen arm: a case report

**DOI:** 10.1186/1752-1947-8-22

**Published:** 2014-01-27

**Authors:** Monica Kidd, Vina Broderick

**Affiliations:** 1 Department of Family Medicine, Faculty of Medicine, University of Calgary, 3330 Hospital Drive NW, Calgary, Alberta T2N 4N1, Canada; 2 Discipline of Family Medicine, Memorial University of Newfoundland, St John’s A1B 3V6, Canada

**Keywords:** Paget–Schröetter syndrome, Subclavian stenosis, Subclavian vein thrombosis, Upper extremity deep vein thrombosis (UEDVT)

## Abstract

**Introduction:**

Subclavian vein thrombosis is a rare but potentially fatal condition that most often occurs iatrogenically or in the context of malignancy. Here we report the case of an active, healthy 32-year-old woman who presented with subtle findings of arm pain, paresthesias and skin changes of acute onset and was subsequently diagnosed with upper extremity deep vein thrombosis and subclavian stenosis, and was started on a course of oral antithrombotics.

**Case presentation:**

A 32-year-old right-handed Caucasian woman presented to her family medicine clinic with left shoulder pain and numbness along her ipsilateral forearm and hand, as well as subtle swelling of the affected limb. Initially diagnosed with medial epicondylitis, she was later diagnosed with subclavian thrombosis caused by Paget–Schröetter syndrome.

**Conclusion:**

Presentations such as these are often attributable to soft-tissue injuries that resolve with rest and sometimes physiotherapy. Subclavian thrombosis was a highly unexpected diagnosis in this case; however, family physicians must remain vigilant in considering rare causes of common clinical presentations which could cause patients significant morbidity if left undiagnosed.

## Introduction

Nearly one-quarter of adult Canadians attend a physician for musculoskeletal (MSK) disorders each year [1], making MSK complaints one of the most common reasons people seek primary care. Most of these cases are benign, so it is understandable for a primary care physician to provide reassurance and symptomatic relief to many patients without exhaustive investigation. However, it is important to remain vigilant for the rare sinister cause of a seemingly benign presentation. Here we report the case of a healthy 32-year-old woman who, after a several-week history of pain and paresthesias, developed a swollen discolored arm. A duplex ultrasound confirmed the presence of a clot in her subclavian vein, a subsequent computed tomography (CT) scan showed subclavian vein stenosis, and venography performed in neutral and in stress positions confirmed bilateral thoracic outlet obstruction. Subclavian vein thrombosis is a rare condition that most often occurs in the context of central venous catheters, surgery or malignancy but, as we describe here, can also have primary causes such as anatomic anomalies and Paget–Schröetter syndrome (so-called “effort thrombosis”) [2]. As with lower extremity deep vein thrombosis (DVT), upper extremity DVTs (UEDVTs) can be fatal or debilitating if untreated; important complications include pulmonary embolism and post-thrombotic syndrome [2-6]. This case reminds us that it is important for family physicians to maintain a wide differential in presentations of seemingly benign MSK complaints.

## Case presentation

A 32-year-old right (R)-handed Caucasian woman presented to her family medicine clinic with a several-week history of left (L) shoulder pain and numbness along her ipsilateral forearm and fifth finger, exacerbated by elbow extension against resistance. She was initially diagnosed with medial epicondylitis and treated with a non-steroidal anti-inflammatory drug; no differential diagnosis was noted by the original physician but it would have included conditions that can lead to brachial plexopathy, including trauma, compressive tumor, cervical radiculopathy, and spinal stenosis.

**Figure 1:**
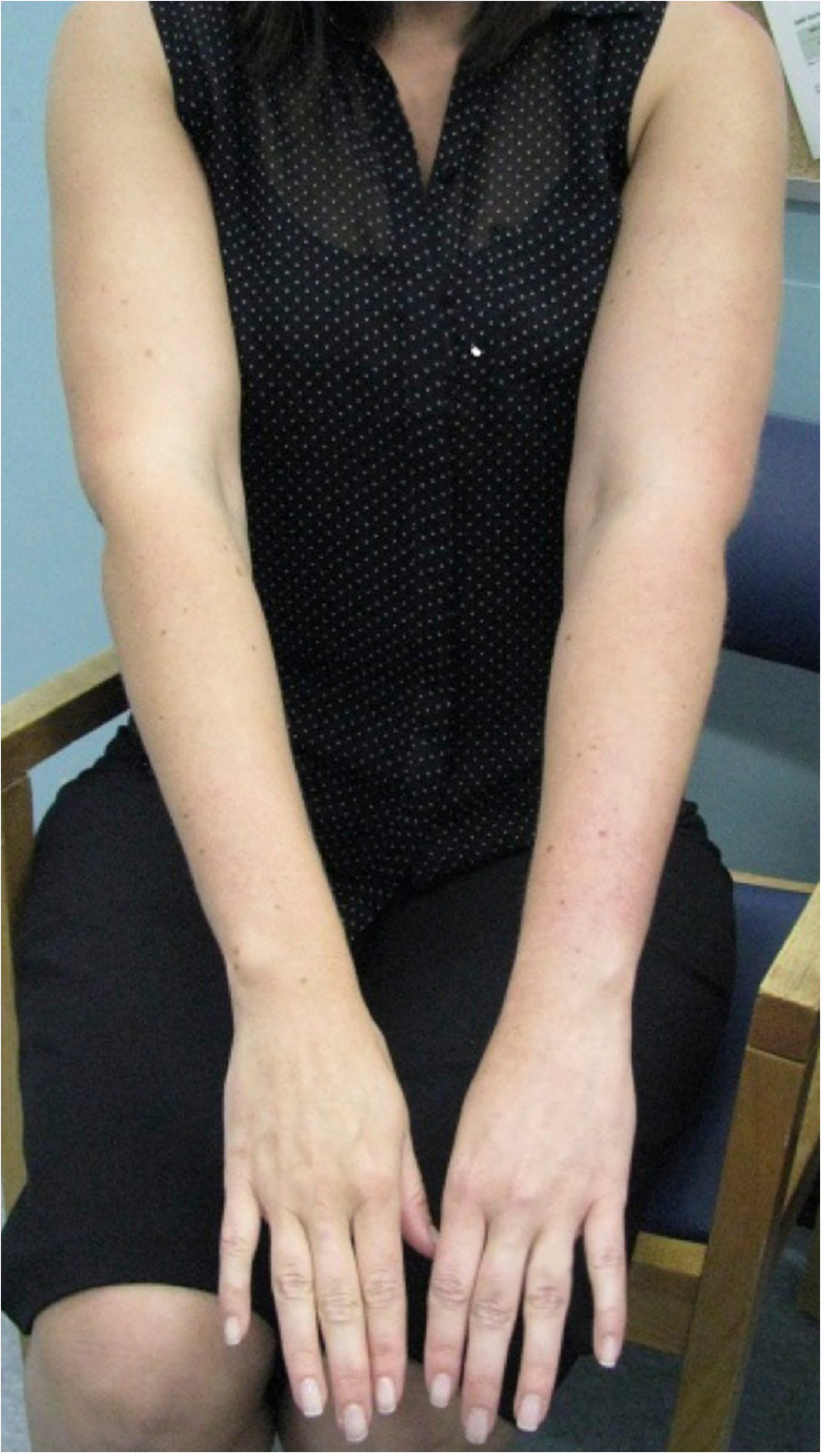
Subtle arm swelling and discoloration associated with subclavian thrombosis.

 When she awoke the following morning, her L arm had become swollen and discolored, so she returned to the clinic. On examination, her L arm was subtly swollen (L upper arm circumference 33cm and 31.5cm on R; L lower arm circumference 23cm and 21cm on R) with a similarly subtle purplish discoloration over the dorsal aspect of her distal L forearm (Figure 1). Both limbs were warm, and radial pulses and capillary refill in her fingers were normal bilaterally. The range of motion in her shoulders, elbows and wrists was normal bilaterally, and deep tendon reflexes at her elbows and wrists were intact. She displayed numbness over her dorsal and ventral L forearm, as well as the lateral aspect and palm of her L hand, but was neurologically intact on the R. She was afebrile and normotensive. Her chest was clear and her heart sounds were normal. She had been previously well, was a non-smoker and had nothing in her history to suggest a Pancoast tumor. She was involved in regular cross-training exercises and had intentionally lost approximately 40kg over the previous 3 years, giving her a body mass index of 25.8. She worked in an office and was engaged to be married in the near future. Her only medication was an oral contraceptive pill (OCP) containing drospirenone.

X-rays of her chest, shoulder and elbow were normal; duplex ultrasound showed subclavian vein thrombosis, even though this can be a challenging diagnosis to make because ultrasound visualization of the area is difficult and the subclavian vein cannot be compressed [7]. She knew of no personal or family history of venous thromboembolic disease. Her OCP was discontinued because we were concerned the clot could be hormone-related, and we referred her to hematology. Although current guidelines do not include evaluation for thrombophilia in the decision of whether or how to treat venous thrombosis [7], hereditary risk factors for thrombosis do affect length of treatment and sometimes monitoring [8], so the standard hypercoagulable screen available in our center was ordered. The screen showed she was negative for lupus anticoagulant, and that her levels of antithrombin, protein C and protein S were all normal; neither did she carry Factor V Leiden or prothrombin allele mutations. She was immediately started on a 3-month course of anticoagulation, beginning with low-molecular-weight heparin injections and a vitamin K antagonist, according to current guidelines (Grade 1B evidence) [9]; she was also prescribed a compression sleeve, although there is Grade 2C evidence against this. Her heparin was discontinued when her international normalized ratio (INR) became therapeutic.

A subsequent CT scan with intravenous contrast injected on the R (contralateral side) showed stenosis of her R subclavian vein from the thoracic inlet to its origin with her superior vena cava. There was no lung pathology. Unfortunately, characterization of the affected side was not possible with this study and was not repeated. She was referred to vascular surgery. A venogram was ordered there and it demonstrated bilateral obstruction of her subclavian veins, with significant stenosis on the L (ipsilateral) side, and complete obstruction on the R (contralateral) side with stress maneuvers, consistent with bilateral thoracic outlet syndrome and resolved Paget–Schröetter syndrome on the L. She was offered bilateral rib resection and at the time of writing is considering her options.

## Discussion

An informal survey of physicians at our family practice teaching clinic revealed only one other case of subclavian thrombosis in the last 16 years, suggesting that in our patient population such presentations are rare. The annual incidence of UEDVT is estimated to be three per 100,000 persons [7], therefore MSK disorders are a much more common cause of arm pain and paresthesias. The majority of UEDEVTs (80%) are secondary to the use of central venous catheters and pacemakers, or to conditions such as malignancy, surgery, trauma, immobilization, OCP use, pregnancy or ovarian hyperstimulation syndrome; only 20% are believed to have primary causes related to anatomical abnormalities (such as a cervical rib or subclavian stenosis) or so-called “effort thrombosis” (Paget–Schröetter syndrome) [10]. It is not uncommon for patients who present with unilateral UEDVT to have bilateral narrow thoracic outlets [5].

Although surgical decompression and venous angioplasty may be considered for thoracic outlet obstruction [4,10,11], the typical treatment for primary subclavian vein thrombosis is oral anticoagulation only [9]. In a recent case–control study in the Netherlands, 45 patients with the condition were treated with: (i) oral anticoagulation only, or (ii) thrombolysis followed by anticoagulation, or (iii) thrombolysis followed by transaxillary rib resection. The study showed that although patients undergoing thrombolysis with or without surgery had less pain, swelling and fatigue in the affected limb at 6 weeks, all patients had the same quality of life at a mean follow-up of 57 months; furthermore, rates of recurrence were similarly low (five instances of recurrence across the three groups) [4]. Given similar outcomes for each of the treatments, anticoagulation alone would seem prudent in many cases; in fact, recent guidelines favor anticoagulation only over thrombolysis plus anticoagulation (Grade 2C) [9].

Our patient presented with a 3-week history of shoulder pain and paresthesias limited to the ulnar distribution, followed by an acutely swollen and discolored arm plus paresthesias in the ulnar, radial and median distributions. Initially, our differential diagnosis included cellulitis, lymphedema, and neoplastic compression of the subclavian vein. She had no fever or obvious site of infection to suggest cellulitis; she had had no previous surgery or previous episodes of swelling to suggest lymphedema; and she had no risk factors and displayed no constitutional symptoms to suggest a malignancy. A duplex ultrasound confirmed subclavian thrombosis, and an X-ray and CT ruled out both tumor and a cervical rib as causes for the swelling. CT showed subclavian stenosis on her unaffected side, and venography confirmed bilateral thoracic outlet obstruction with stress maneuvers consistent with Paget–Schröetter syndrome.

Although it seemed this primary anatomical anomaly was the probable cause of our patient’s clot, we remained uncertain as to the contribution, if any, of her OCP or her cross-training. She was motivated to continue with exercise, and all of her physicians supported this decision once her INR became therapeutic. Her vascular surgeon supported this approach. She was also considering starting a family soon, and we advised her that she may require anticoagulation with low-weight molecular heparin during future pregnancies. We discontinued her compression sleeve at about 1 month, once her swelling and discoloration had resolved, but vascular surgery recommended she resume using it for at least 12 months, perhaps longer, in order to prevent problems with potential post-phlebitic syndrome.

## Conclusion

MSK complaints comprise a large majority of presentations to the family physician, and most often they are soft-tissue injuries that resolve with rest and physiotherapy. Occasionally, more sinister causes are uncovered that demand treatment and can result in long-term morbidity. UEDVT is a rare cause of arm pain and sensory changes, but should remain part of the differential in the context of limb swelling and discoloration.

## Consent

Written informed consent was obtained from the patient for publication of this case report and accompanying images. A copy of the written consent is available for review by the Editor-in-Chief of this journal.

## Abbreviations

CT: Computed tomography; DVT: Deep vein thrombosis; INR: International normalized ratio; L: Left; MSK: Musculoskeletal; OCP: Oral contraceptive pill; R: Right; UEDVT: Upper extremity deep vein thrombosis.

## Competing interests

The authors declare that they have no competing interests*.*

## Authors’ contributions

MK saw the patient at her second presentation, made the diagnosis and initiated her treatment. She also performed the literature review and analysis, and contributed to writing the manuscript. VB provided many aspects of the patient’s medical history, continues to see her in follow up and continues to coordinate her investigations. She contributed to writing the manuscript. Both authors read and approved the final manuscript.
